# Heading for New Shores: Projecting Marine Distribution Ranges of Selected Larger Foraminifera

**DOI:** 10.1371/journal.pone.0062182

**Published:** 2013-04-19

**Authors:** Anna E. Weinmann, Dennis Rödder, Stefan Lötters, Martin R. Langer

**Affiliations:** 1 Steinmann-Institut für Geologie, Mineralogie und Paläontologie, Universität Bonn, Bonn, Germany; 2 Zoologisches Forschungsmuseum Alexander Koenig, Bonn, Germany; 3 Institut für Biogeographie, Universität Trier, Trier, Germany; Bangor University, United Kingdom

## Abstract

The distribution of modern symbiont-bearing larger foraminifera is confined to tropical and subtropical shallow water marine habitats and a narrow range of environmental variables (e.g. temperature). Most of today's taxa are restricted to tropical and subtropical regions (between 30°N and 30°S) and their minimum temperature limits are governed by the 14 to 20°C isotherms. However, during times of extensive global warming (e.g., the Eocene and Miocene), larger foraminifera have been found as far north as 50°N (North America and Central Europe) as well as towards 47°S in New Zealand. During the last century, sea surface temperatures have been rising significantly. This trend is expected to continue and climate change scenarios for 2050 suggest a further increase by 1 to 3°C. We applied Species Distribution Models to assess potential distribution range changes of three taxa of larger foraminifera under current and future climate. The studied foraminifera include *Archaias angulatus*, *Calcarina* spp., and *Amphistegina* spp., and represent taxa with regional, superregional and global distribution patterns. Under present environmental conditions, *Amphistegina* spp. shows the largest potential distribution, apparently due to its temperature tolerance. Both *Archaias angulatus* and *Calcarina* spp. display potential distributions that cover currently uninhabited regions. Under climate conditions expected for the year 2050, all taxa should display latitudinal range expansions between 1 to 2.5 degrees both north- and southward. The modeled range projections suggest that some larger foraminifera may colonize biogeographic regions that so far seemed unsuitable. *Archaias angulatus* and *Calcarina* spp. also show an increase in habitat suitability within their native occurrence ranges, suggesting that their tolerance for maximum temperatures has yet not been fully exploited and that they benefit from ocean warming. Our findings suggest an increased role of larger foraminifera as carbonate producers and reef framework builders in future oceans.

## Introduction

Larger, symbiont-bearing foraminifera are marine protists that are abundant in tropical and subtropical reef and shelf-regions of the world's oceans. They are major contributors to carbonate production and play an important role in the formation and stability of global reefs [1], [2]. Larger symbiont-bearing foraminifera are most abundant in warm, oligotrophic waters [3], [4]. They are frequently associated with scleractinian corals due to their corresponding environmental requirements. Previous studies show that distribution patterns of larger foraminifera are mainly controlled by ocean temperature and nutrient content [3], [4]. In our study, we applied Species Distribution Models (SDMs) on three taxa of larger foraminifera: *Archaias angulatus* (Fichtel & Moll, 1798), *Calcarina* spp. d’Orbigny, 1826 and Amphistegina spp. d’Orbigny, 1826. *Archaias angulatus* is a regionally distributed species, which occurs almost exclusively within the western Atlantic Ocean and is among the dominant species in the shallow waters of the Caribbean Sea. Calcarinid foraminifera exhibit a superregional distribution within the eastern Indian Ocean and the western Pacific [3] and are prominent contributors within the center of marine biodiversity [3], [5]. Amphisteginids are circum-globally distributed and represents one of the most widely distributed and ubiquitous taxa of symbiont-bearing foraminifera.

Within the last decades, SDMs have become an important tool to identify and evaluate potential distributions of taxa based on their specific environmental tolerances [6], [7]. Most studies applying SDMs have focused on terrestrial plants and animals, but available modeling techniques are applicable for marine species as well [8], [9]. Such SDMs have been developed for a variety of different taxa [8], [10]–[14]. However, compared to terrestrial organisms, the field of modeling marine species is still scarce [9], [13], [15]. Reasons for this include the complexity of oceanographic interactions [9], the three dimensionality of the system [8] and the lack of environmental variables in grid-formats which are compatible with the established modeling software. Here, we present global scale SDMs for three selected taxa of larger foraminifera based on remote-sensing datasets.

An important application of SDMs is the prediction of likely range changes of taxa in the context of anthropogenic global change [16]. During the 20^th^ century, global ocean temperatures have risen by 0.7°C [17]. Temperatures will further rise by a mean value of 1.5°C until 2050 [18]. This has already led to major impacts on marine biotas [17], [19]. Scientists are in concert that rapid climate change affects and increases the extinction risk for biota around the globe [20] but that such changes have frequently occurred in the past [21]. The latter mentioned aspect may indicate the capability of taxa to rapidly adapt to environmental changes [22]. On the other hand, extant species show range shifts as an adaption to changing climate rather than they adapt to climate change by evolution [23].

The fossil record provides extensive evidence that larger foraminifera have been abundant and widely distributed during times of particularly warm climate intervals [2], [3], [24]. Climate warming was usually accompanied by biogeographic range extensions towards higher latitudes [24]. Here, we apply SDMs on three taxa of larger symbiont-bearing foraminifera to assess the magnitude and direction of distributional range changes based on a future climate scenario for the year 2050.

## Materials and Methods

### Species records and environmental data

A total of 189 records of the three foraminiferal taxa were available through previous research on the biogeography of larger foraminifera (Table S1). Of these, 123 records were available for *Amphistegina* spp., 35 for *Archaias angulatus*, and 31 for *Calcarina* spp. For a detailed listing of the sample stations and their references, see Table S1. Even though relatively few species records are available for each *Archaias angulatus* and *Calcarina* spp., these records cover the known extremes of the species' environmental requirements and therefore are here tentatively employed in model building. All of these records were situated within unique grid cells of 4 km×4 km. The accuracy of the coordinates was assessed with DIVA-GIS 7.1.7 [25] by comparing information provided with the records with locality (land shape, bathymetry; see below).

Information on oceanographic data was obtained via remote sensing. For sea-surface temperature (SST) we used daytime (maximum temperature in °C) and nighttime (minimum temperature in °C) monthly averages from 1985 to 2007 with a resolution of 4 km (2.5 arcmin). In this study, we used AVHRR Pathfinder Version 5.2 (PFV5.2) data, obtained from the US National Oceanographic Data Center and GHRSST (http://pathfinder.nodc.noaa.gov). The PFV5.2 data are an updated version of the Pathfinder Version 5.0 and 5.1 collections described in [26]. Annual mean sea-surface temperatures were calculated from the monthly averages with DIVA-GIS. Although the studied foraminiferal species display a benthic life style, SST can be used here, as it is a proxy for the general water temperature. As abiotic factors we used annual mean images quantifying salinity [psu], nitrate concentrations [ µmol/l], phosphate concentrations [ µmol/l] and silicate concentrations [ µmol/l] from the World Ocean Atlas 2005 in 0.25° and 1° resolution [27]. The datasets were converted to raster format compatible with GISs and were, if necessary, interpolated with ArcGis 9.3 (ESRI), using a bivariate smoothing spline of available oceanographic values and land shape as limiting barrier.

**Figure 1:**
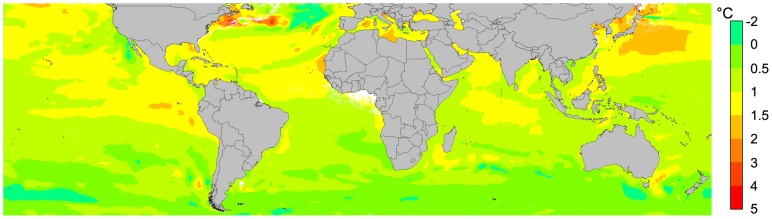
Expected sea surface temperature variations for 2050. The model is based on the IPCC scenario A1B assuming a world of rapid economic growth and a balance between fossil-intensive and renewable energy technologies [18], [28].

To estimate likely range changes under a future climate scenario, we used the AquaMaps dataset [28], which describes variations in annual mean sea-surface temperatures as expected in 2050 (Figure 1), based on the IPCC climate change scenario A1B [18]. The local differences between the present and the future temperature layers were calculated for each grid-cell and added to our own data set following the delta approach, which has been successfully applied in previous studies [29].

### Computation of SDMs

We used Maxent 3.3.3k [30] to model the potential distributions of the foraminiferal species and to project them into geographic space. Maxent is a presence-only SDM method (generating pseudo-absences) and uses a grid-based machine-learning algorithm following the principles of maximum entropy [31]. For an overview on the operating mode of Maxent and the interpretation of its output see [15], [32]. Ideally, pseudo-absence records used for SDM training cover those areas potentially colonizable for the target species [33]. Since the habitats of larger benthic foraminifera are exclusively located in reefs and shelf regions of the world's oceans, all environmental grids were clipped in order to use only shelf regions for the modeling process. The grids were overlain by a bathymetry-grid derived from the ETOPO1 Global Relief Model [34], leaving only the coastal regions between 0 and 1350 m water depth. A total of 10,000 random background points were automatically selected by Maxent from this area.

The logistic output format with suitability values ranging from 0 (unsuitable) to 1 (optimal) was used [35], where the probability of presence at sites with typical conditions is approximately 0.5 [32]. Maxent allows for model testing by calculation of the Area Under the Curve (AUC), referring to the Receiver Operation Characteristic (ROC) curve [30]. The occurrence records were split into training (70%) and test samples (30%) and for each taxon we computed 50 single SDMs and the average predictions across all replicates were used for further processing. Being non-parametric, this method is recommended for ecological applications [36]. Values of AUC range from 0.5 (i.e. random) for models with no predictive ability better than random to 1.0 for models giving perfect discrimination between presence and pseudo-absence records. According to the classification of Swets [37], AUC values>0.9 describe “very good”,>0.8 “good” and>0.7 “useful” discrimination ability. The continuous probability surfaces of the SDMs were subsequently converted into presence/absence maps using the “minimum training presence” as a threshold representing the minimum predicted value assigned to any of the training localities [30]. Those areas with a probability of occurrence above this threshold can be interpreted as regions with comparable environmental conditions as at the native occurrences of the taxa.

SDMs were refined in a two-step clipping process in order to avoid a biased relation between the variables temperature and ion concentrations (previous modeling attempts resulted in an unlikely high contribution of salinity in expense of other variables). In the first SDM, we used only SST which was subsequently projected on the future climate scenario. The second SDM was built on salinity, nitrate, phosphate and silicate. In doing so, we achieved a climate-model based on temperature which was later overlain and clipped by a habitat-model based on ion concentrations. The editing of the climate model was performed with DIVA-GIS.

## Results

**Figure 2:**
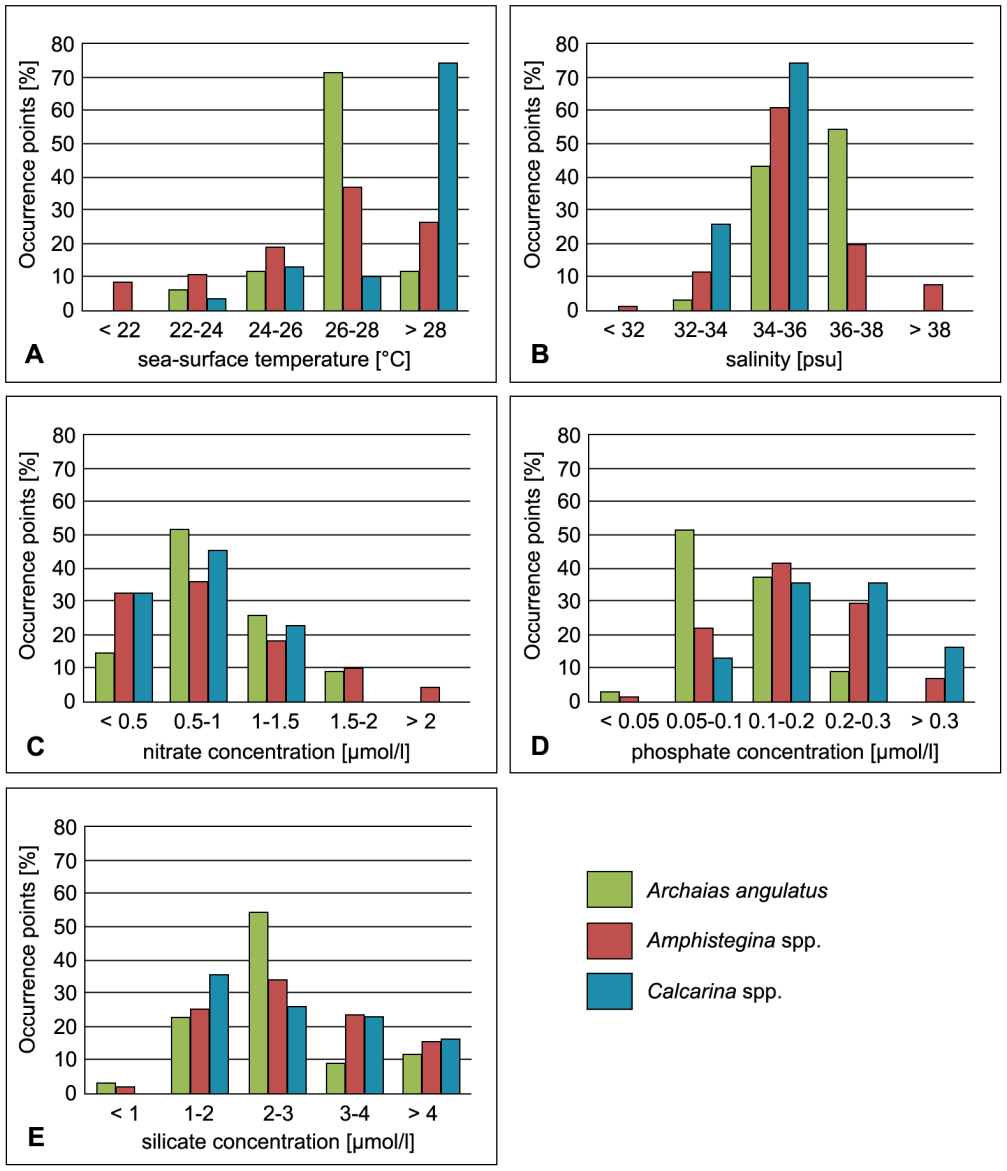
Relationship of spatial distribution and environmental variables. (A) Occurrence points of *Archaias angulatus* (green), *Amphistegina* spp. (red) and *Calcarina* spp. (blue) are plotted against sea surface temperature values at the respective location; (B) Occurrence points of *Archaias angulatus* (green), *Amphistegina* spp. (orange) and *Calcarina* spp. (blue) are plotted against salinity values at the respective location; (C) Occurrence points of *Archaias angulatus* (green), *Amphistegina* spp. (red) and *Calcarina* spp. (blue) are plotted against nitrate values at the respective location; (D) Occurrence points of *Archaias angulatus* (green), *Amphistegina* spp. (red) and *Calcarina* spp. (blue) are plotted against phosphate values at the respective location; (E) Occurrence points of *Archaias angulatus* (green), *Amphistegina* spp. (red) and *Calcarina* spp. (blue) are plotted against silicate values at the respective location.

We received “good” to “useful” AUC values for SDMs based on SST of *Archaias angulatus* (AUC_training_: 0.87; AUC_test_: 0.87), *Amphistegina* spp. (AUC_training_: 0.83; AUC_test_: 0.79) and *Calcarina* spp. (AUC_training_: 0.77; AUC_test_: 0.74). The evaluation of the variable contribution with regard to ion concentration in the habitat-models showed that for *Archaias angulatus*, phosphate concentration (with 80%) had the highest explanatory power. For both *Amphistegina* spp. and *Calcarina* spp. nitrate concentration was deemed the most useful variable (63 and 64%, respectively). The correlation of occurrence records and the modeled variables are depicted in Figure 2.

Under current climate conditions, all three taxa displayed distributional ranges quite consistent with their native occurrences. In the following, the potential distributions of *Archaias angulatus* and *Amphistegina* spp. in the Atlantic Ocean, the distributions of *Calcarina* spp. and *Amphistegina* spp. in the Indopacific as well as changes with future climate are described in detail.

### *Archaias angulatus*

**Figure 3:**
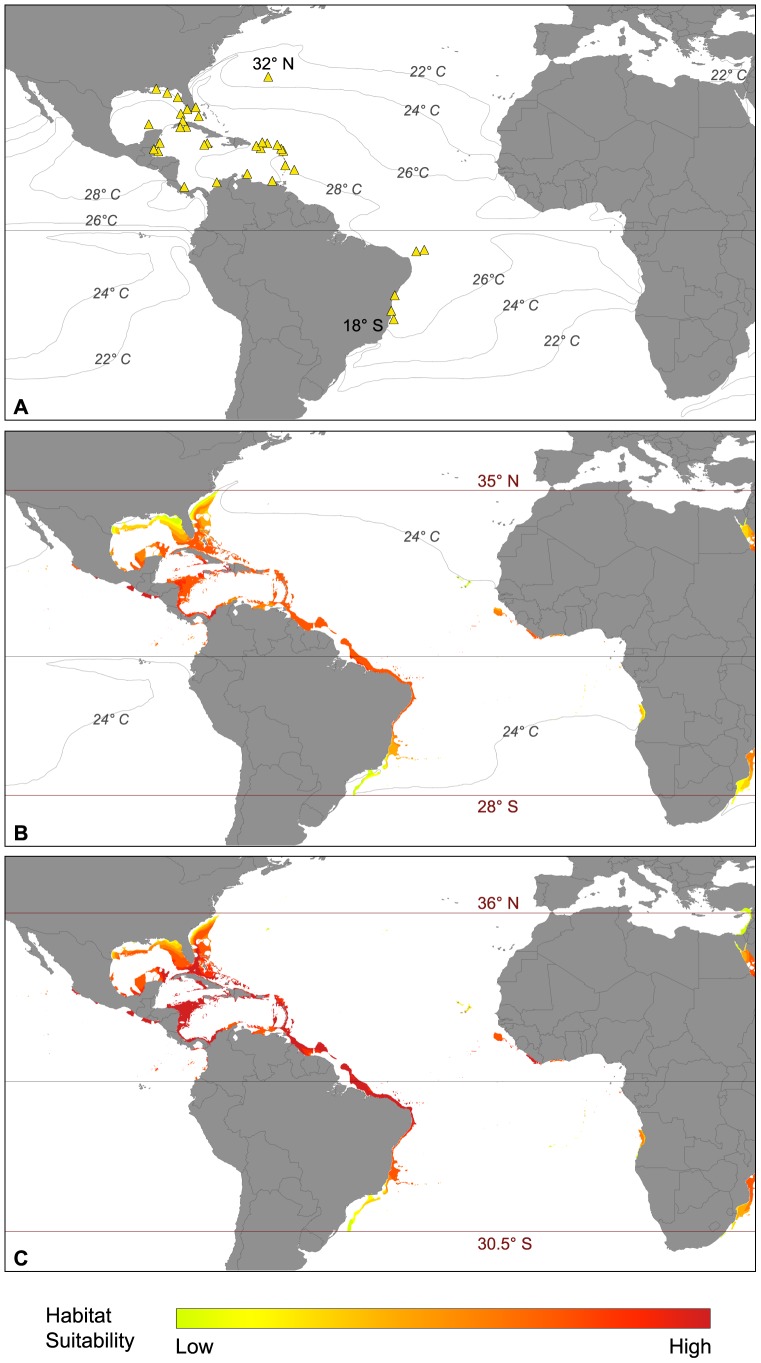
Biogeographic distribution of *Archaias angulatus* in the Atlantic Ocean. (A) Actual distribution and major isotherms (triangles: occurrence records used in the modeling process); (B) potential distribution under present climate conditions and corresponding isotherms; (C) potential distribution under future climate conditions.

The native distribution of *Archaias angulatus* is in the western Atlantic Ocean, mainly within the Caribbean (Figure 3A). Its latitudinal range extends from 32°N in Bermuda to 17.5°S in Abrolhos, Brazil (Table S1). The SDM based on current climatic conditions (Figure 3B) displayed a potential distribution, which is overall well presenting the species' actual realized distribution as suggested by the occurrence points. In the North, the model suggested habitat suitability up to 35°N, with an area of relatively low suitability along the northern part of Florida, Georgia and South Carolina. In the South, the model predicted the potential distribution of *Archaias angulatus* to extend to 28°S, south of Rio de Janeiro in central Brazil. Areas, which were predicted to be most suitable for the occurrence of *Archaias angulatus*, were situated within the core region of this species, especially the central Caribbean and also the northern coast of Brazil (except for the Amazon Delta). The model suggested further suitable areas for *Archaias angulatus* under present climate conditions along the Pacific Coast of Central and South America from 30°N in northern Mexico to 2°N in Colombia. Suitable settling areas for *Archaias angulatus* may include habitats in the eastern Atlantic, especially off Guinea, Liberia and Ivory Coast. Furthermore, the future SDM predicted suitable conditions for *Archaias angulatus* in the Cape Verde Islands, providing a potential stepping stone for a possible colonization of West Africa.

Under future climatic conditions (Figure 3C), areas of highest habitat suitability for *Archaias angulatus* were predicted to expand, resulting in an area-wide coverage of the Caribbean Sea from southern and eastern Florida (30°N) to Bahia in Brazil (11°S). Potentially suitable areas in the Pacific and eastern Atlantic Ocean would increase. The latitudinal range of *Archaias angulatus* would expand both north- and southwards. The predicted range expansion under future climate in the north comprised 1 degree (approximately 100 km - up to 36°N), resulting in suitable environmental conditions for *Archaias angulatus* along the coast of North Carolina. In addition, the SDM predicted that the southern range limit will lie at around 30.5°S, in southern Brazil, which would indicate a range expansion of approximately 2.5 degrees (about 270 km). The model predicted a southward range expansion following the shelf margin.

Environmental parameters included in our SDM indicated that more than 82% of all occurrence records of *Archaias angulatus* were located in regions that exceed 26°C of annual mean water temperature (Figure 2A). The lowest SST associated with *Archaias angulatus* was 23.1°C and the highest temperature was 28.4°C. The distribution of the taxon correlated with normal marine salinity conditions (between 34 and 38 psu; Figure 2B) and it preferred lower nitrate, phosphate and silicate values (Figures 2C-2E).

### *Amphistegina* spp

Amphisteginid foraminifera display some of the widest latitudinal extensions among the larger foraminifera analyzed to date. They exhibit a true circum-global distribution and are present in all tropical and subtropical oceans. The modeling approach applied here focuses on the Atlantic, Mediterranean and Indo-Pacific Ocean regions, in order to provide better comparison with *Archaias angulatus* and *Calcarina* spp.

#### Atlantic Ocean and Mediterranean Sea

**Figure 4:**
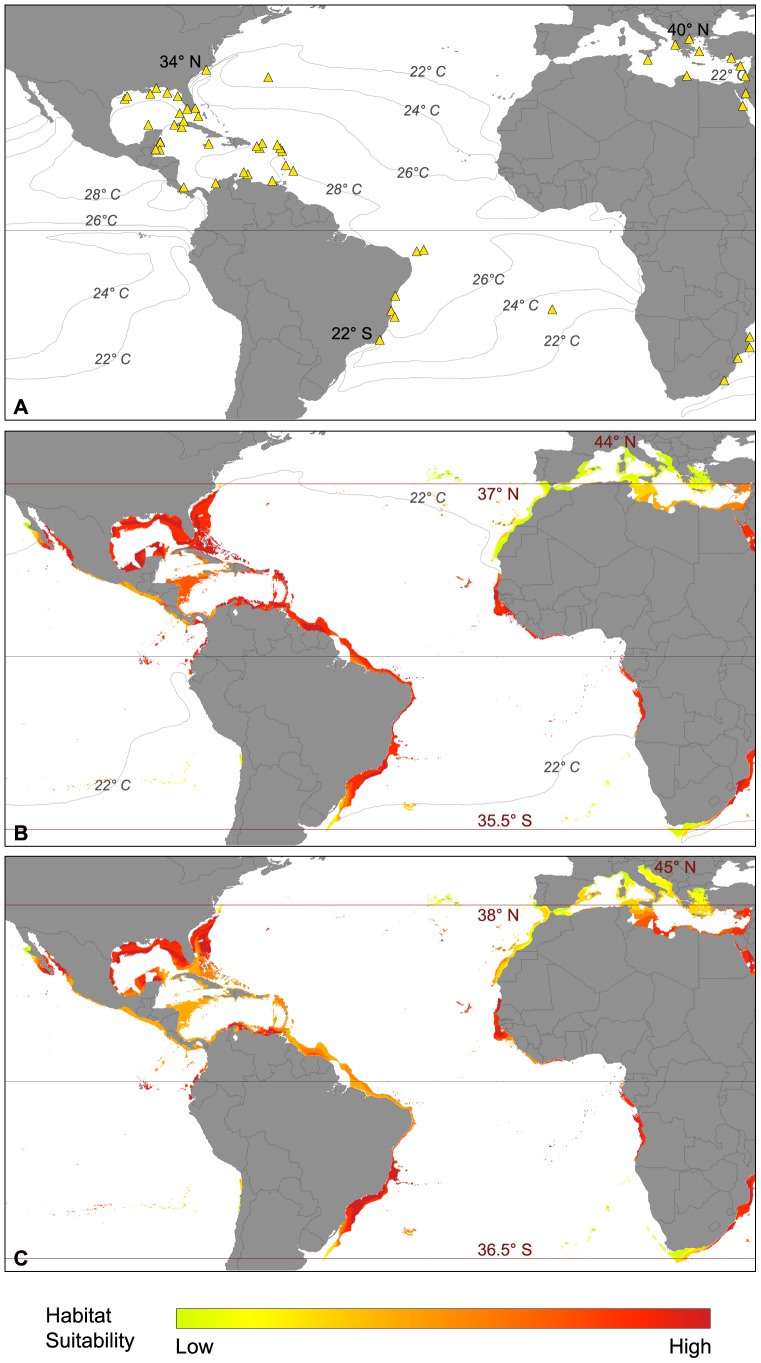
Biogeographic distribution of *Amphistegina* spp. in the Atlantic Ocean. (A) Actual distribution and major isotherms (triangles: occurrence records used in the modeling process); (B) potential distribution under present climate conditions and corresponding isotherms; (C) potential distribution under future climate conditions.

*Amphistegina* spp. is widely distributed within the western Atlantic Ocean (Figure 4A). The latitudinal range currently covers an area of 56 degrees ranging from 34°N in North Carolina to 22°S at Cape Sao Tome, Brazil (Table S1). The taxon has also been reported from Saint Helena and the Canary Islands. In addition, amphisteginid foraminifera successfully invaded the Mediterranean Sea after the opening of the Suez Canal. Their current distribution in the Mediterranean extends to 40°N ([15] and references therein). Under current climatic conditions (Figure 4B), the SDM suggested a potential distribution, which covers the entire shelf region of the western Atlantic and Caribbean Sea from 37°N in North Carolina to 35.5°S in southern Brazil and northern Uruguay. Again, at the southern border, suitable habitats appeared to be limited to offshore shelf regions. Habitat suitability within the latitudinal range was generally high, with most appropriate regions situated around 10 to 20°N and S. On the Pacific side of the American continent, *Amphistegina* spp. may find suitable habitats from 31°N in Baja California to 3°S in Ecuador. The model further suggested acceptable habitat suitability under present climate conditions on the Peruvian-Chilean border, within the Galapagos Archipelago and to the Easter Islands, where *Amphistegina* spp. has actually been recorded (Table S1). In the eastern Atlantic Ocean, the SDM predicted highly suitable areas on the Cape Verde Islands and the western African coast from 17°N in southern Mauretania to 15°S in Angola. In the Mediterranean Sea, the present climate model suggested adequate habitat suitability in the eastern Mediterranean (Egypt, Israel, Syria and Turkey with highest values) and also towards the central Mediterranean.

Under future climatic conditions (Figure 4C), *Amphistegina* spp. may still find suitable habitat within its today's realized distribution in the western Atlantic Ocean. However, habitat suitability was predicted to decrease slightly within lower latitudes, especially in the Caribbean Island Arc and the western part of the Caribbean Sea as well as the northern coast of Brazil. However, no habitat loss is to be expected. Towards the southern border, the model predicted that habitat suitability increases. The future model also suggested a latitudinal range expansion of the taxon, with 1 degree in the North and South respectively (approximately 100 km). Along the Pacific coast of North America amphisteginids are not likely to expand their potential latitudinal distribution range. Off western Africa, appropriate settling areas off Liberia and the Ivory Coast decreased slightly. Within the Mediterranean Sea, habitat suitability in the eastern and southern part was suggested to drastically increase under future climate conditions and the SDM predicted further suitable areas within the western Mediterranean. This may result in a latitudinal range expansion up to 45°N.

#### Indo-Pacific Ocean and Red Sea

**Figure 5:**
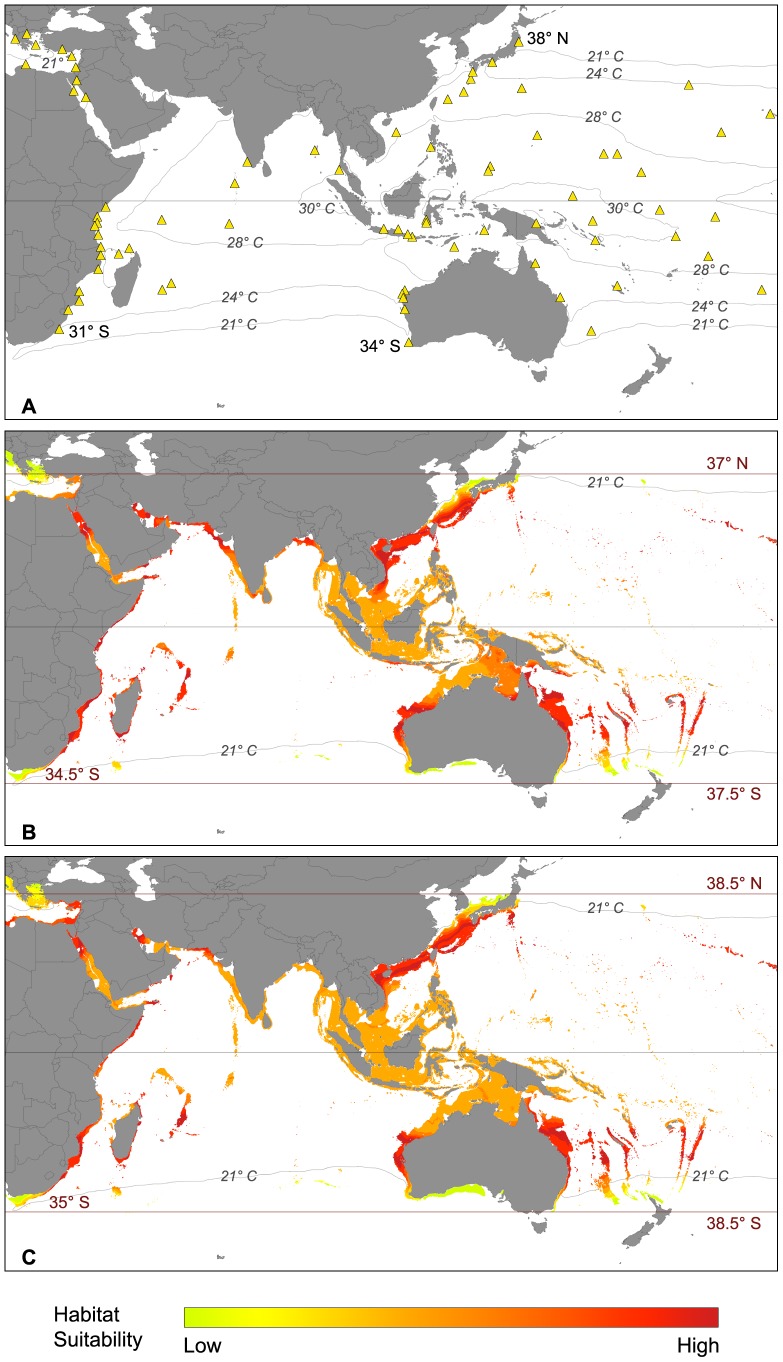
Biogeographic distribution of *Amphistegina* spp. in the Indo-Pacific Ocean. (A) Actual distribution and major isotherms (triangles: occurrence records used in the modeling process); (B) potential distribution under present climate conditions and corresponding isotherms; (C) potential distribution under future climate conditions.

Within the Indo-Pacific Ocean (Figure 5A), the native distribution of *Amphistegina* spp. covers a latitudinal range of 72 degrees ranging from 38°N in Sendai, eastern Japan to 34°S in western Australia (Table S1). The taxon is well distributed along the remote islands of the Pacific, as well as on all major islands within the Indian Ocean. It is a major faunal contributor along the coasts of India and eastern Africa (up to 31°S) and also within the Red Sea and the Persian Gulf (Table S1). The SDM built under current climate conditions (Figure 5B) suggested a potential distribution that covers all of the native occurrences of *Amphistegina* spp. The latitudinal expansion of the potential distribution ranged from 37°N in eastern Japan to 37.5°S in Australia and 34.5°S in South Africa. Habitat suitability ranged from medium to high within the latitudinal range of *Amphistegina* spp. with the highest values predicted within the western Indian Ocean as well as the areas 33°N to 15°N and 13°S to 32°S in the western Pacific. Regions between 15°N and 13°S generally showed lower habitat suitability under present climate conditions, but the conditions were still within the apparently acceptable range for *Amphistegina* spp. Areas of low habitat suitability were identified at the northern and southern range limits of the potential distribution in South Africa, Japan and Australia.

Under future climatic conditions (Figure 5C), the SDM suggested similar habitat suitability for *Amphistegina* spp. at lower latitudes of the Indo-Pacific Ocean. Conditions in Samoa and south of Tonga towards the islands north of New Zealand became more suitable for the species of *Amphistegina*. However, New Zealand itself is not predicted to be suitable for colonization. In East Africa, along the Seychelles and within the Persian Gulf region, suitability was suggested to locally decrease slightly, but without habitat loss. Within the Red Sea, appropriate suitability values decreased in the north but increased in the South. The latitudinal range of *Amphistegina* spp. is likely to expand under future climate conditions towards the north up to 38.5°N to eastern Japan, resulting in a potential northward migration of approximately 150 km. Towards the south, the SMD suggested a latitudinal range expansion of 0.5 degrees (∼50 km) to 35°S in South Africa and about 1 degree towards 38.5°S (100 km) in eastern Australia. Along the southern part of South Africa and southern Australia, an additional longitudinal expansion was predicted.

The temperature range of *Amphistegina* spp. went from 15.3°C to 29.6°C (Figure 2A). The taxon preferred lower concentrations of nitrate, phosphate and silicate as well as normal marine salinity conditions (Figures 2B to 2E).

### *Calcarina* spp

**Figure 6:**
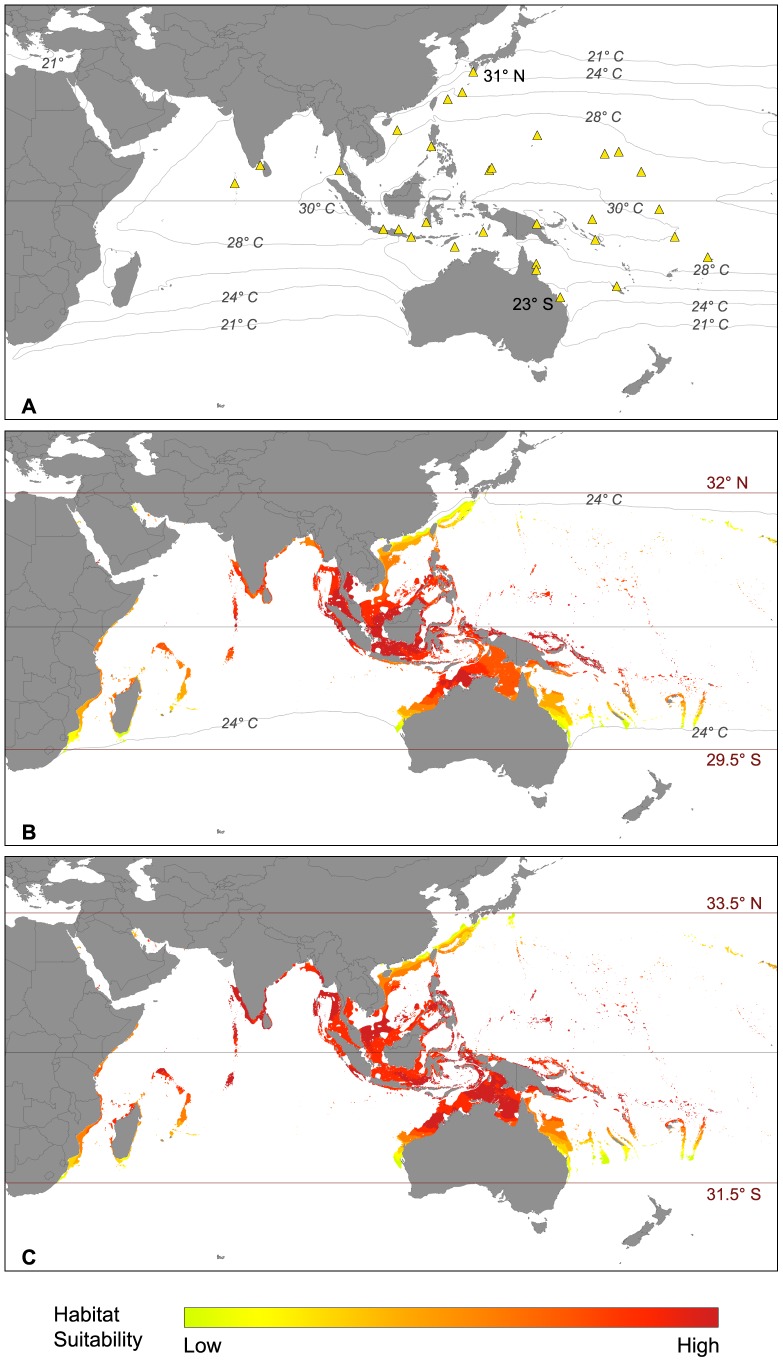
Biogeographic distribution of *Calcarina* spp. in the Indo-Pacific Ocean. (A) Actual distribution and major isotherms (triangles: occurrence records used in the modeling process); (B) potential distribution under present climate conditions and corresponding isotherms; (C) potential distribution under future climate conditions.

The native distribution of *Calcarina* spp. is within the Indopacific region ranging from the Maldives in the west to Samoa in the east (Figure 6A). Its latitudinal range covers an area of 54 degrees from 31°N in southern Japan to 23°S in Eastern Australia (Table S1). Under present climate conditions (Figure 6B), the SDM predicted suitable habitats between 32°N in southern Japan to 29.5°S in Australia. The areas with highest habitat suitability lay within the shelf regions of central and southern Asia. Habitat suitability continuously decreased towards higher latitudes both in the north as well as the south. In the West Pacific, potentially colonizable areas with suitable environmental conditions for *Calcarina* spp. were predicted to include French Polynesia and, to a lesser degree, Tonga and the Hawaiian Islands. Under present climate conditions, potential habitats with high suitability within the Indian Ocean included the coasts of India and Myanmar, the Maldives and the Seychelles. Medium and lower values were recorded by the SDM for Madagascar and the coast of East Africa. The model also considered the southern Red Sea as highly appropriate for colonization but suitability decreased towards the northern part of the Red Sea.

The SDM projected into future climate conditions (Figure 6C) suggested an increase of habitat suitability for *Calcarina* spp. within its core region. Areas of higher suitability were expanding towards the north and the south and the latitudinal range of the genus was also predicted to increase. In the north, the model suggested suitable environmental conditions up to 33.5°N in southern Japan, resulting in a predicted migration of 1.5 degree (approximately 150 km). In the south, suitable habitats were predicted to expand towards 31.5°S in eastern Australia, suggesting a latitudinal range extension of up to 2 degrees (about 220 km). Under future climate conditions, latitudinal range shifts were also predicted for the western coast of Australia and the West Pacific. Suitability values in Hawaii were also increased. A general increase in habitat suitability was also predicted for the northern and western Indian Ocean. The Persian Gulf and almost the complete Red Sea were also suggested as highly appropriate for *Calcarina* spp.

*Calcarina* spp. preferred high SST values with more than 70% of the occurrence records correlated with temperatures above 28°C (Figure 2A). The taxon favors normal marine salinity conditions and is relatively tolerant towards increased phosphate concentrations (Figures 2B and 2D). On the other hand, it showed a preference for lower nitrate and silicate values (Figures 2C and 2E).

## Discussion

The SDMs developed in this study for *Archaias angulatus*, *Amphistegina* spp. and *Calcarina* spp. provide new insights into patterns and ecological constraints regulating the biogeography of larger foraminifera. Furthermore, our SDM approach prognosticates possible range expansions of larger foraminifera under predicted future climate, as all models made sufficient predictions of habitat suitability within the native occurrence regions of the three taxa studied (Figures 3, 4, 5, 6).

### Distribution patterns and controlling oceanographic parameters

#### *Archaias angulatus*

For the regionally distributed *Archaias angulatus* in the Atlantic Ocean, the SDM developed under present climate conditions suggested high suitability of habitats within the Caribbean region and the northern coast of Central and South America (Figure 3B). Suitability values at the northern and southern borders were low and increased successively towards the Bahamas, Florida and Bahia (Brazil) where *Archaias angulatus* is known to occur in high abundances [38]–[40]. In many studies on Caribbean foraminifera, *Archaias angulatus* is described as a dominant faunal constituent [41], [42]. This in turn suggests that the environmental conditions within its native range are close to its optimum, both concerning abiotic parameters and biotic interaction.

Environmental parameters included in our SDM indicated that 82% of all occurrence records of *Archaias angulatus* were located in regions that exceed 26°C of annual mean water temperature (Figure 2A). The SDM further suggested that the distribution of the species highly correlated with the 24°C isotherm (Figure 3B). Thus, temperatures between 24°C and 29°C apparently mark the temperature optimum for *Archaias angulatus*. The upper temperature limit is capped by the natural SST limit within the Caribbean and Atlantic Ocean. Other studies within the western Atlantic region also report high abundances of the species at temperatures between 26°C and 29°C [41], [42]. Our model also indicated that the distribution of *Archaias angulatus* correlated with low nutrient levels (Figures 2C to 2E). The species, however, is known to tolerate certain threshold values of nutrients and its carbon fixation ratio by feeding is ten times higher than by symbiotic contribution [43]. *Archaias angulatus* preferred higher salinity conditions than *Amphistegina* spp. (Figure 2B), which has also been reported in other studies [42]. *Archaias angulatus* is common in shallow reef and open shelf sites and has a predominantly epiphytic lifestyle on seagrass (especially *Thalassia testudinum*) and macroalgae [4], [39], [40], [42]. It is most abundant in back reef and inner shelf environments [38], [41], [44].

The northern distribution range of *Archaias angulatus* along the coast of the United States is limited by the deflection of the warm Gulf Stream from the coast. North of Florida, the coastal waters become significantly colder as the Gulf Stream follows the outer continental shelf before turning eastward. The limited suitability within the Gulf of Mexico can be traced back to the higher nutrient levels due to major river runoffs. The southern border of *Archaias angulatus* correlates with the southernmost coral reef formations in the Atlantic Ocean [45], which also mark the southern limit of the South Brazilian faunal province [46]. Towards the south, the warm Brazil current detaches from the coast resulting in coastal waters that are too cold for the establishment of reef-building corals [47] as well as *Archaias angulatus*.

The species could find limited suitable habitats along the Pacific coast of Central America and western Africa. *Archaias angulatus* is of neotropical origin and evolved during the Miocene [48] before the closure of the Central American seaway and the isolation of the Caribbean region from the Pacific realm [49]. Today, it is not reported from the eastern Pacific or the eastern Atlantic with the easternmost expansion of *Archaias angulatus* being known from the Cape Verde Islands [3]. Yet, if it was introduced in these regions (e.g. by anthropogenic transport) it might proliferate as an invasive species. However, large parts of the western coast of Africa are under the influence of upwelling or cold-current systems and provide the shelf regions with nutrient-rich waters [50]. Furthermore, true coral reefs are absent from the eastern Atlantic Ocean [47], thus limiting the settlement of the reef-dwelling organisms.

#### *Amphistegina *spp

*Amphistegina* spp. is a 'true' circum-global taxon, which is present in almost all subtropical and tropical shelf regions of the world's oceans [3]. It is a major faunal contributor to foraminiferal assemblages in shallow water habitats as well as an important contributor to carbonate production [2], [4], [15]. The species has the widest biogeographic range observed among the modern larger foraminifera covering a total north-south range from more than 50 degrees of latitude (from 34°N to 22°S) in the Atlantic Ocean to more than 70 degrees of latitude (from 38°N to 34°S) in the Indo-Pacific Ocean [3]. This is consistent with the maximum latitudinal range proposed for larger foraminifera [51]. According to our SDM, its potential distribution under current climate covered a latitudinal range over 70 degrees in both regions (Figures 4B and 5B). In the Atlantic Ocean, the northern and southern range limits of *Amphistegina* spp. were previously described [52]. Within the Gulf of Mexico, the Caribbean and the coast of northern South America, habitat suitability in our SDM was generally high. In the eastern Atlantic Ocean, the SDM suggested high habitat suitability along the western coast of Africa as well as on the Cape Verde Islands and Saint Helena, from which *Amphistegina* spp. has previously been reported [53], [54]. It is absent from the Senegal and most of the Gulf of Guinea [55], [56] as well as the northwestern coast of Africa and the Canary Islands [53]. However, environmental conditions would support potential colonization, if the taxon was introduced in these areas. Findings of *Amphistegina* spp. from the Gulf of Guinea [56] and the Congolese Shelf between Gabon and Angola [57] are probably relict faunas. Barruseau et al. [58] described rich *Amphistegina gibbosa* layers from the Senegal to the Congo, which are dated back to 12,000 years before present. The isolated Quaternary recordings of *Amphistegina* spp. from this region were considered to represent short-term invaders that have entered the area during the warmer interglacial periods with present day colder waters preventing recolonization [3].

In the Mediterranean, our SDM suggested range expansions towards the northwestern Mediterranean, which was also shown by contemporary studies in this region [15, Weinmann et al., submitted].

Within the Indo-Pacific Ocean, the northernmost occurrences of *Amphistegina* spp. are reported from 38°N in Sendai, Japan which is just outside the potential distribution of the taxon (Figure 5B). According to the SDM, the coastal areas of the western Indian Ocean suited better for the requirements of *Amphistegina* spp. than those of the Indo-Malayan region. *Amphistegina* spp. has long been known to be an important faunal contributor along the eastern coast of Africa from the Red Sea to South Africa (Table S1).

Among the taxa of larger foraminifera studied, *Amphistegina* spp. displayed the widest temperature tolerance with a range of more than 14 degrees of annual mean sea surface temperature (Figure 2A). In the Caribbean and eastern Atlantic the 22°C isotherm appears to restrict the highly suitable areas of the potential distribution (Figure 4B). In the Indo-Pacific Ocean the distribution limit of *Amphistegina* spp. is restricted by the 21°C isotherm. In the Mediterranean, SST values were within the lower temperature range of the taxon *Amphistegina* spp. is known to have the widest temperature tolerance within the group of larger foraminifera [59] with a minimum limit of 14°C [3], [60]. Recent observations within the Mediterranean suggested amphisteginids may survive short-term exposure to even lower temperatures (13.7°C) [15], [61].

In the western Atlantic Ocean, *Amphistegina* spp. is abundant in fore-reef environments and along the upper reef slope. It is also common on carbonate platform margins [38], [41]. The same is true for the Indo-Pacific Ocean where *Amphistegina* spp. is abundant on slope regions down to 130 m of water depth and on hard-ground substrates like reef rubble [4], [52], [62]–[65]. Since large-scale coral reefs are absent from the Mediterranean Sea, *Amphistegina* spp. often dwells as an epiphyte on seagrass or algae.

Similar to *Archaias angulatus*, the distributional limit of *Amphistegina* spp. in the North Atlantic is determined by the warm waters of the Gulf Stream and its interactions with colder waters along the East coast of North America. Within the Gulf of Mexico and along the northern coast of South America, the distribution of amphisteginid foraminifera is severely limited by the runoff from major rivers (Mississippi, Amazon and the Orinoco). The southern distribution limit of *Amphistegina* spp. is controlled by the warm Brazil Current, which detaches from the coast at approximately 12°S and continues to flow southward along the continental margin of Brazil [50]. At 33°S, the confluence zone between the warm Brazil Current and the cold Malvinas Current begins, resulting in a rapid decline of sea surface temperature and salinity. The absence of coral reefs south of the Abrolhos Archipelago parallels the absence of amphisteginids in southern Brazil.

Along the eastern coast of the Pacific, the cold California and Humboldt Currents constitute severe limitations to the potential distribution of amphisteginid foraminifers. Both currents are governed by global current and upwelling systems [50]. High habitat suitability was predicted for the western coast of Mexico and Baja California. Within the latter region, only Quaternary records for *Amphistegina* spp. exist [66]. The Isthmus of Panama, which closed 3.5 million years ago, acts as the main dispersal barrier between the Caribbean and the eastern Pacific region [49]. Furthermore, coral reefs are extremely limited on the western coast of the Americas [47].

Within the Indo-Pacific, the distribution ranges along the east coast of southern Africa is controlled by the warm Agulhas Current, which deflects from the coast towards the south. Along the coasts of Somalia and Oman the suitability for colonization is limited by seasonal upwelling of the Somali Current [50]. Southern Japan is influenced by the warm Kuroshio Current, which extends subtropical regions further to the North [67] and thus allows the colonization of eastern and central Japan.

Along Australia, both the western and the eastern coastlines to 34°S were considered highly suitable by the SDM for the settlement of *Amphistegina* spp. This was apparently due to the warm Leeuwin Current in the West and the East Australian Current in the East [68]. Many of the remote islands of the central and Eastern Pacific Ocean show abundant occurrences of *Amphistegina* spp. [3]. Within this area, a longitudinal dispersal of faunal elements is facilitated by the Equatorial Counter Current. The genus *Amphistegina* first occurred during the Eocene and has since seized a distributional range that includes the entire circum-tropical belt [49]. The most prominent example for the adaptability and expansive capabilities of *Amphistegina* spp. is currently within the Mediterranean, where the taxon has established itself extremely well since the reopening of the connection between the tropical Red Sea and the Mediterranean [15].

#### *Calcarina* spp

For *Calcarina* spp., which are restricted to the eastern Indian Ocean and the western Pacific [3], [69], the SDM predicted highest habitat suitability within the tropical region of South-East Asia (Figure 6B). Habitat suitability decreased to medium and low values towards the northern (32°N) and southern (29.5°S) borders of the potential distribution for the genus *Calcarina*.

Calcarinid foraminifera are prominent faunal contributors to the southern Ryukyu Islands, including Okinawa [62]. Interestingly, the SDM suggested that the environmental conditions in the Central Pacific Ocean (e.g., Hawaii) were favorable for calcarinid foraminifera but the taxon is absent from regions east of Samoa [3], [69], [70]. This may be the result of restricted dispersal capabilities and the lack of potential stepping stones [3]. *Calcarina* spp. is also absent from the western Indian Ocean and the Red Sea, where suitability values were indicated by our model to range between low and medium values. In the Central Indian Ocean, environmental conditions were suggested to be highly advantageous. Recent reports from the Maldives' Archipelago suggest that the species is currently extending its natural range westwards [71]. This record marks the westernmost occurrence of the taxon, since *Calcarina* spp. has not been reported from the Chagos Archipelago, which is located in the Central Indian Ocean [72].

In our SDMs, *Calcarina* spp. preferred high SST values with more than 70% of all occurrence records in areas characterized by annual mean temperatures of≥28°C (Figure 2A). Their occurrence within the Indo-Pacific region appears to correspond to the 24°C isotherm (see Figure 6B) and highest habitat suitability values were observed at SST temperatures above 28°C. Various studies reported high abundances of *Calcarina* spp. in areas with annual mean temperatures between 26°C and 30°C [3], [64], [70]. From the data currently available, *Calcarina* spp. has its optimal temperature requirements in the warmest reef areas of modern oceans. Calcarinid and related foraminifera (*Neorotalia calcar*, *Pararotalia spinigera*) have a reportedly high tolerance to elevated sea-surface temperatures [73] and some of them may even tolerate diel temperature fluctuations that range between 28° and 36°C [Langer (unpubl. data)]. Laboratory studies revealed that heat-stress induced bleaching in calcarinids is less distinct than in other larger foraminifera [74]. In addition, particularly dense populations of living calcarinid foraminifera were frequently observed in warm water pools around Raja Ampat (Papua New Guinea) at temperatures above 31°C [Langer (unpubl. data)]. This indicates that calcarinids may tolerate even higher temperatures than previously suggested.

*Calcarina* spp. appeared to prefer lower nitrate and silicate concentrations (Figures 2C and 2E). However, higher tolerance of nutrients has been reported from previous studies [62], [69]. *Calcarina* spp. also preferred salinity values between 32 and 36 psu (Figure 2B), which are consistent with previous studies [75]. The taxon is commonly restricted to shallow waters (< 40 m) and is predominantly associated with macroalgae and algal turf [4], [64], [65], [69], [70], [76]. Due to its morphology, *Calcarina* spp. is able to attach itself on phytal substrates and is adapted to high energy environments [64], [69], [75]. The thick walls may provide shelter from UV radiation, resulting in a higher tolerance to strong illumination conditions compared to other larger foraminifera and allows for high abundances of this taxon in shallow water [62], [65], [69].

In the western North Pacific the distribution limit of *Calcarina* spp. is controlled by the northward flowing warm Kuroshio Current that provides subtropical conditions in southern Japan [67]. The absence of the taxon north of 31°N correlates with the limitation of coral reefs to 30°N [47], which results in the absence of reef environments that are often associated with *Calcarina* spp. To the south, the Leeuwin Current and the East Australian Current govern the temperature regimes of Western and Eastern Australia, thus providing suitable conditions for *Calcarina* spp. [68]. Coral reefs around Australia extend to approximately 30°S [47] on both sides of the continent. *Calcarina* spp. has neither been recorded from the atolls in southeastern Australia [77], nor from Lord Howe Island at 31°S [76]. Instead, *Baculogypsina* spp., which has comparable environmental requirements, has been reported from Lord Howe Island [76]. Because the distribution of *Calcarina* spp. is related to the 24°C isotherm, we suggest that its absence from this region is related to temperature rather than to the presence of *Baculogypsina* spp. *Calcarina* spp. is absent from the Central and Eastern Pacific Ocean. The same trend has been observed in the Indian Ocean, where *Calcarina* spp. is also widely absent. The Equatorial Counter Current within the Indian Ocean seems to be a major inhibitor of the westward expansion of *Calcarina* spp. [69]. Furthermore, *Neorotalia calcar*, which occupies almost the same microhabitats as *Calcarina* spp., is very abundant in the western Indian Ocean, especially in the tropical regions of eastern Africa [Weinmann (unpubl. data)]. *Calcarina* spp. evolved in the late Miocene or Pliocene [48], [49], [78] and has the shortest geological record of all taxa discussed in this study.

### Interspecific comparisons

A comparison between the potential distributions of *Archaias angulatus* and *Amphistegina* spp. in the Caribbean and *Amphistegina* spp. and *Calcarina* spp. in the Indo-Pacific Ocean revealed some differences in habitat suitability values. In the Caribbean regions, that were considered to be most suitable for *Amphistegina* spp., the SDM showed lower suitability values for *Archaias angulatus* (Figure 3B and 4B). On the other hand, *Archaias angulatus* displayed higher suitability values in areas that were deemed less suitable for *Amphistegina* spp. The same trend could be observed in the Indo-Pacific Ocean, where *Calcarina* spp. showed lower suitability values than *Amphistegina* spp. in certain regions and vice versa (Figures 5B and 6B). All taxa studied here live in the warm shallow coastal, reefal and shelf habitats. As such one would expect comparable suitability values and somewhat uniform potential range expansions. However, the taxa exhibit specific microhabitat preferences [4], [38]–[42], [52], [62], [64], [65], [70], [76]. In addition, the study presented here shows large-scale, taxon-specific preferences for temperature, salinity and other ion concentrations (see Figure 2). Microhabitat specific preferences of individual taxa are therefore considered to be controlling agents of future range expansion capabilities.

The SDMs revealed that the potential distributions of the studied species not only included the native occurrence regions of the taxa, but also showed potentially suitable areas under current and future conditions. As such, the suitability suggests areas that might be affected by future species invasion. Because foraminifera are among the most prolific reefal faunal elements, invasions may affect ecosystem functioning that ultimately affects the native biota. As has been previously shown [15], the invasion of amphisteginids into the Mediterranean Sea impacts the faunal composition, biodiversity and carbonate productivity and results in substantial faunal alterations (see also [79]).

### Future range expansions due to climate change and possible implications

Analysis of all taxa studied here revealed that they are likely to expand their biogeographic ranges with future climate warming (Figures 3C, 4C, 5C and 6C). Increased habitat suitability for individual taxa suggested that this may lead to higher abundances, potentially impacting the structure and composition of native faunal assemblages. The results are consistent with observations of range expansions during the last decades reported for various marine and terrestrial taxa [23], [80].

The range shifts recorded occur at a much faster rate than previously assumed [23]. Range shifting and species invasions due to rising sea surface temperatures in the Mediterranean have been documented for numerous taxa [81] including foraminifers ([15] and references therein). Larger foraminifera have wider latitudinal ranges and increased abundances during exceptionally warm periods in Earth's history [2]–[3], [24], [52]. During the Eocene, *Amphistegina* spp. had a particularly wide distribution ranging from 48°N on the Eastern coast of the USA to 36°S in New Zealand during the Eocene [52]. During the Miocene, the extent of *Amphistegina* spp. covered the area from 50°N in the Vienna Basin and Poland [52] to 37°S in Eastern Australia and 47°S in New Zealand [52]. Quaternary records from South Australia revealed the presence of *Amphistegina* spp. at 35°S, presumably during interglacials [82]. *Archaias angulatus* has been recorded from France during the Pliocene [3]. Calcarinids significantly increased in abundance during the early Pliocene [83], which was correlated with the stronger influence of the West Pacific Warm Pool during this time due to the collision of Australia with Asia [67], [83]. Larger foraminifera are well adapted to high sea surface temperatures and they seem to be less affected by temperature rises than corals [2], [84], which suffer from significant symbiont loss or “bleaching” [2], [4], [85], [86]. Bleaching also occurs in larger foraminifers but this is probably associated with increased UV radiation rather than temperature stress [4], [86]. In modern oceans, larger foraminifers are important contributors to the ocean carbonate production [1], [2]. Under future climate conditions, larger foraminifera may again represent one of the major groups of carbonate producers of shallow water reefal structures [2], [84], [85], as has been the case during the particularly warm time intervals of the Carboniferous, Permian, the Upper Cretaceous and the Eocene [24], [84], [87], [88]. The abundance and carbonate production of larger foraminifera under warmer climates is particularly well documented for the Eocene, where nummulitid buildups form giant shoals, banks and reefal structures in tropical ramp and platform settings. In addition, the Paleocene-Eocene maximum is correlated with the “Larger Foraminifera Turnover” within the Tethys, represented by increased evolution and diversification as well as widespread accumulations of larger foraminifera, especially nummulitids [89], [90].

A renaissance of larger foraminifera as prominent producers of reefal carbonate in a warmer future climate, however, is currently challenged by oceans' rising acid levels [17], [84], [85]. The rapid change in ocean chemistry affects carbonate producers on all levels and is projected to decrease by 0.3–0.4 pH units in the year 2100 [18]. The potential effect of ocean acidification on tests of larger foraminifera has been recently studied in laboratory experiments, where foraminifera were exposed to CO_2_ levels of up to 2000 ppmv [91]–[93]. While some species showed limitations in growth rates after exposure to∼1000 ppmv [91], [92], the species relevant to this study (*Calcarina gaudichaudii* and *Amphistegina gibbosa*) appeared to be less affected [92], [93]. McIntyre-Wressnig et al. [93] showed that no lethal effects were observed in *Amphistegina gibbosa* even under 2000 ppmv. This is significantly higher than prognosticated for the year 2100. Ocean acidification is not novel per se and CO_2_ levels during the early Eocene are assumed to be two to three times higher than today [85]. Interestingly, larger nummulitid foraminifera were among the dominant carbonate producers during that time [85]. In the following, temperature and CO_2_ levels decreased during the Oligocene and corals re-emerged as the most important reef-building taxa [85]. The environmental niche constraints of the larger foraminifera presented here in addition to the findings from the fossil record and acidification experiments support the hypothesis, that some larger foraminifera may be beneficiaries of a warmer future climate.

## Conclusions

Species distribution modeling of selected larger symbiont-bearing foraminifera to predict potential range shifts under current and future climates lead to the following principal conclusions:

1. Distribution patterns of larger foraminiferal taxa like *Archaias angulatus*, *Amphistegina* spp. and *Calcarina* spp. are mainly governed by temperature and restricted by range-specific temperatures.
2. The environmental envelope of each taxon, as defined by the current occurrence records and environmental variables from the same sites, account for the distribution patterns on a global scale.
3. Modeled projections reveal that under present climatic conditions, potential distribution ranges are not fully utilized. Identification of suitable range shifts by the SDM may be useful to alert us to the extent and magnitude of future climate change impacts.
4. Under future climate conditions (model for 2050), all of the studied taxa exhibit latitudinal range expansions and increases in habitat suitability. This suggests that at least some larger symbiont-bearing foraminifera have the capability to exploit new suitable habitats under altered climates and may ultimately benefit from global warming.
5. The range expansions predicted by the Species Distribution Model are in agreement with previous paleontological findings that larger foraminifera have succeeded at the expense of other organism when ocean temperatures have risen to extremes during warm phases of Earth history (e.g., Eocene, Upper Cretaceous).

## Supporting Information

Table S1 **Occurrence records and references for *Archaias angulatus*, *Amphistegina* spp. and *Calcarina* spp. used in this study.**
(DOCX)
