# Supplementary material for: Heading for New Shores: Projecting Marine Distribution Ranges of Selected Larger Foraminifera
Source: PLoS One. 2013 Apr 19;8(4):e62182. doi: 10.1371/journal.pone.0062182 (PMC3631173; doi:10.1371/journal.pone.0062182)
Supplement: Table S1 — Occurrence records and references for Archaias angulatus, Amphistegina spp. and Calcarina spp. used in this study. (DOCX) [file pone.0062182.s001.docx]

**Table S1**. Occurrence records and references for *Archaias angulatus*, *Amphistegina* spp. and *Calcarina* spp. used in this study.

(A.: *Amphistegina* spp.; C.: *Calcarina* spp.; Aa.: *Archaias angulatus*)

| **Sites** | **Taxa** | **References** |
| --- | --- | --- |
| ***Australia*** |  |  |
| West Australia | A. | [1] |
| Bateman Bay | A. | [2] |
| Norwegian Bay | A. | [2] |
| Exmouth | A. | [3] |
| Shark Bay | A. | [3] |
| Heron Island | A., C. | [3] |
| Lord Howe Island | A. | [3] |
| Green Island | C. | [4] |
| Lizard Island | A., C. | [3] |
| Sahul Shelf | A., C. | [3] |
| ***Pacific and Southeast Asia*** |  |  |
| Hawaii | A. | [3] |
| Easter Islands | A. | [3] |
| Pitcairn Island | A. | [3] |
| Rarotonga | A. | [3] |
| New Caledonia | A., C. | [3] |
| Tahiti | A. | [3] |
| Scilly | A. | [3] |
| Samoa | A., C. | [3] |
| Funafuti | A., C. | [3, 5] |
| Honiara Bay | A., C. | [3] |
| Solomon Islands | A., C. | [3] |
| Phoenix Island | A. | [3] |
| Onotoa Atoll | A., C. | [3] |
| Kapinagamaringi Atoll | A. | [3] |
| Majuro Atoll | A., C. | [6, 7] |
| Palau | A., C. | [3] |
| Kayangel Atoll | A., C. | [8] |
| Enewetak Atoll | A., C. | [8] |
| Marshall Islands | A., C. | [3] |
| Mariana Islands | A., C. | [3] |
| Johnston Atoll | A. | [3] |
| Midway Islands | A. | [3] |
| China Sea | A., C. | [3] |
| Bali | A., C. | [3, 9] |
| Indonesia | A., C. | [3] |
| Java | A., C. | [3] |
| Jakarta | A., C. | [3] |
| Madang | A., C. | [10] |
| Sulawesi | A., C. | [11] |
| Kabaena | A. | [3] |
| Thailand | A., C. | [3] |
| Philippines | A., C. | [3] |
| Ishigaki | A., C. | [12] |
| Okinawa | A., C. | [3, 13] |
| Ogasawara | A. | [3] |
| Tokara Island | A. | [3] |
| Kagoshima | A., C. | [3] |
| Tanabe Bay | A. | [14] |
| Sendai | A. | [3] |
| ***Indian Ocean*** |  |  |
| Shelley Beach | A. | [15] |
| Inhaca | A. | [16, 17] |
| Inhambane | A. | [18] |
| Bazaruto | A. | [19] |
| Mafamete | A. | [15] |
| Pemba | A. | [15] |
| Quirimbas | A. | [20] |
| Mafia Island | A. | [15] |
| Zanzibar Island | A. | [15] |
| Pemba Island | A. | [15] |
| Kenya | A. | [3] |
| Somalia | A. | [21] |
| Réunion | A. | [22] |
| Mascareigne Island | A. | [3] |
| Mayotte | A. | [3] |
| Glorioso | A. | [23] |
| Chagos Island | A. | [3] |
| Seychelles | A. | [3] |
| Maldives | A., C. | [24] |
| India | C. | [4] |
| Coshin | A. | [3] |
| Andamanen Islands | A. | [3] |
| ***Atlantic*** |  |  |
| Rio de Janeiro | A. | [25] |
| Abrolhos | A., Aa. | [26, 27, 28] |
| Recife de Fora | A., Aa. | [29] |
| St. Helena | A. | [3] |
| Salvador | A., Aa. | [3] |
| Atoll das Rocas | A., Aa. | [27, 28] |
| Fernando de Noronha | A., Aa. | [3, 28] |
| ***Caribbean*** |  |  |
| Panama | A., Aa. | [30] |
| Catargena | A., Aa. | [3] |
| Venezuela | A., Aa. | [3] |
| Curacao | A., Aa. | [27, 31, 32] |
| Aruba | A. | [3] |
| Barbados | A., Aa. | [3] |
| St. Lucia | A., Aa. | [27, 33] |
| Guadeloupe | A., Aa. | [34] |
| Belize | A., Aa. | [35] |
| British Honduras | A., Aa. | [34] |
| Antigua | A., Aa. | [27, 31] |
| St. Kitts | A., Aa. | [34] |
| S Puerto Rico | A., Aa. | [34] |
| Jamaica | A., Aa. | [34] |
| Ponce | A. | [34] |
| SW Puerto Rico | A., Aa. | [34] |
| St. Martin | A., Aa. | [34] |
| Puerto Rico | Aa | [3] |
| Jamaica | A., Aa. | [34] |
| San Juan | A., Aa. | [34] |
| Yucatan | A., Aa. | [36] |
| Cuba | Aa | [3] |
| Batabano | A., Aa. | [34] |
| Campeche Bank | A., Aa. | [37] |
| W Cuba | A. | [34] |
| Havanna | A., Aa. | [34] |
| Bahamas | A., Aa. | [3] |
| Tortugas | A., Aa. | [37] |
| Cape Romano | A., Aa. | [37] |
| Florida | A., Aa. | [3] |
| NE Gulf of Mexico | A., Aa. | [37] |
| Stetson Bank | A. | [37] |
| Claypile Bank | A. | [37] |
| Mississippi Delta | A. | [37] |
| NE Gulf of Mexico | A., Aa. | [37] |
| Gulf of Mexico | A., Aa. | [3] |
| Bermuda | A., Aa. | [3] |
| North Carolina | A. | [3] |
| ***Red Sea and Mediterranean*** |  |  |
| Red Sea | A. | [3] |
| Safaga | A. | [3] |
| Eilat | A. | [3] |
| Haifa | A. | [3] |
| Libya | A. | [3] |
| Cyprus | A. | [38] |
| Malta | A. | [38, 39] |
| Antalya Bay | A. | [38] |
| Korthi | A. | [38, 40] |
| Corfu | A. | [38] |
| Kallikratia | A. | [38, 40] |

**References**

1) Li Q, James NP, Bone Y, McGowran B (1999) Palaeoceanographic significance of recent foraminiferal biofacies on the southern shelf of Western Australia: a preliminary study. Palaeogeogr Palaeoclimatol Palaeoecol 147: 101–120.

2) Parker JH (2009) Taxonomy of foraminifera from Ningaloo Reef, Western Australia. Canberra: Association of Australasian Palaeontologists Memoir 36. 810 p.

3) Langer MR, Hottinger L (2000) Biogeography of selected “larger” foraminifera. Micropaleontology 46: 105–126.

4) Lobegeier MK (2002) Benthic foraminifera of the family Calcarinidae from Green Island Reef, Great Barrier Reef province. J Foram Res 32: 201–216.

5) Collen JD, Garton DW (2004) Larger foraminifera and sedimentation around Fongafale Island, Funafuti Atoll, Tuvalu. Coral Reefs 23: 445–454.

6) Fujita K, Osawa Y, Kayanne H, Ide Y, Yamano H (2009) Distribution and sediment production of large benthic foraminifers on reef flats of the Majuro Atoll, Marshall Islands. Coral Reefs 28: 29–45.

7) Osawa Y, Fujita K, Umezawa Y, Kayanne H, Ide Y et al. (2010) Human impacts on large benthic foraminifers near a densely populated area of Majuro Atoll, Marshall Islands. Mar Pollut Bull 60: 1279–1287.

8) Yamano H, Kayanne H, Matsuda F, Tsuji Y (2002) Lagoonal facies, ages, and sedimentation in three atolls in the Pacific. Mar Geol 185: 233–247.

9) Renema W (2003) Larger foraminifera on reefs around Bali. Zool Verh Leiden 345: 337–366.

10) Langer MR, Lipps JH (2003) Foraminiferal distribution and diversity, Madang Reef and Lagoon, Papua New Guinea. Coral Reefs 22: 143–154.

11) Renema W, Troelstra SR (2001) Larger foraminifera distribution on a mesotrophic carbonate shelf in SW Sulawesi (Indonesia). Palaeogeogr Palaeoclimatol Palaeoecol 175: 125–146.

12) Sugihara K, Masunaga N, Fujita K (2006) Latitudinal changes in larger benthic foraminiferal assemblages in shallow-water reef sediments along the Ryukyu Islands, Japan. Island Arc 15: 437–454.

13) Saraswati PK, Seto K, Nomura R (2004) Oxygen and carbon isotopic variation in co-existing larger foraminifera from a reef £at at Akajima, Okinawa, Japan. Mar Micropaleontol 50: 339–349.

14) Uchio T (1965) Foraminiferal assemblages in the vicinity of the Seto Marine Biological Laboratory, Shirahama-Cho, Wakayama-Ken, Japan. Publications of the Seto Marine Biological Laboratory 15: 399-417.

15) Langer MR, Weinmann AE, Lötters S, Bernhard JM, Rödder D (2013) Climate-driven range extension of *Amphistegina* (Protista, Foraminiferida): Models of current and predicted future ranges. PLoS One 8: e54443. doi:10.1371/journal.pone.0054443.

16) Moura AR (1965) Foraminiferos da Ilha da Inhaca. Revista dos Estudos gerais universitarios de Mozambique, Série 2, Ciencias Biológicas e Agrano´micas 2: 1–74.

17) Perry CT (2003) Coral reefs in a high-latitude, siliciclastic barrier island setting: reef framework and sediment production at Inhaca Island, southern Mozambique. Coral Reefs 22: 485–497.

18) Braga JM (1961) Foraminíferos da costa de Moçambique. Publicaçoñes do Instituto de Zoologia ‘‘Dr. Augusto Nobre’’ Faculdade de ciências do Porto 77: 1–208.

19) Langer MR, Thissen JM, Makled WA, Weinmann AE (2013) The foraminifera from the Bazaruto Archipelago (Mozambique). N Jb Geol Paläontol Abh 297: 155–170.

20) Heron-Allen E, Earland A (1915) The foraminifera of the Kerimba Archipelago (Portuguese East Africa). Part II. Trans Zool Soc London 20: 543–794.

21) Pignatti J, Frezza V, Benedetti A, Carbone F, Accordi G, Matteucci R (2012) Recent foraminiferal assemblages from mixed carbonate-siliciclastic sediments of southern Somalia and eastern Kenya. Boll Soc Geol It 131: 47–65.

22) Montaggioni LF (1981) Les associations de foraminifères dans les sédiments de récifaux de l’archipel des Mascareignes (Océan Indien). Ann Inst Oceanograph 57: 41–62.

23) Battistini R, Gayet J, Jouannic C, Labracherie M, Peypouquet JP, et al. (1976) Etude des sédiments de la microfaune des Iles Glorieuses (Canal de Mozambique). Cahiers du Office de la Recherche Scientifique et Technique Outre-mer, Se´rie Geologie 8: 147–171.

24) Parker JH, Gischler E (2011) Modern foraminiferal distribution and diversity in two atolls from the Maldives, Indian Ocean. Mar Micropaleontol 78: 30–49.

25) Todd R (1976) Some observations about *Amphistegina* (foraminifera). Progress in Micropaleontology, Selected papers in honor of Prof. Kiyoshi Asano, Micropaleontology Press Special Publication: 382–394.

26) Araújo HAB, Machado AJ (2008) Benthic foraminifera associated with the South Bahia coral reefs, Brazil. J Foram Res 38: 23–38.

27) Weinmann AE, Langer MR (2010) Diversity patterns of larger foraminifera within the tropical Western Atlantic Ocean. FORAMS 2010, International Symposium on Foraminifera, Abstracts: 200.

28) Weinmann AE (2009) Benthic shallow-water foraminifera from Fernando de Noronha and Abrolhos, Brazil: A comparative study between two archipelagos and biogeographic analysis of larger foraminifera within the tropical Western Atlantic Ocean. Unpublished Diploma Thesis, Rheinische Friedrich-Wilhelms-Universität Bonn, Germany. 172 p.

29) Bruno RLM, Araújo HAB, Machado AJ (2009) Análise de assembléias de foraminíferos no sedimento superficial do Recife de Fora, região sul da Bahia. Rev Bras Geoci 39: 599–607.

30) Havach SM, Collins LS (1997) The distribution of recent benthic foraminifera across habitats of Bocas del Toro, Caribbean Panama. J Foram Res 27: 232–249.

31) Hofker J (1964) Foraminifera from the tidal zone in the Netherlands Antilles and other West Indian seas. Studies on the Fauna of Curacao and other Caribbean Islands 21: 1–119.

32) Hofker J (1971) The Foraminifera of Piscadera Bay, Curaçao. Studies on the Fauna of Curaçao and other Caribbean Islands 35: 1–57.

33) Sen Gupta BK, Schafer CT (1973) Holocene benthonic foraminifera in leeward bays of St. Lucia, West Indies. Micropaleontology 19: 341–365.

34) Culver SJ, Buzas MA (1982) Distribution of recent benthic foraminifera in the Caribbean region. Smithson Contrib Mar Sci 14: 388 pp.

35) Gischler E, Hauser I, Heinrich K, Scheitel U (2003) Characterization of depositional environments in isolated carbonate platforms based on benthic foraminifera, Belize, Central America. Palaios 18: 236–255.

36) Gischler E, Möder A (2009) Modern benthic foraminifera on Banco Chinchorro, Quintana Roo, Mexico. Facies 55: 27–35.

37) Culver SJ, Buzas MA (1981) Distribution of recent benthic foraminifera in the Gulf of Mexico, Vol. 1. Smithson Contrib Mar Sci 8: 418 pp.

38) Langer MR, Weinmann AE, Lötters S, Rödder D (2012) “Strangers“ in Paradise: Modeling the biogeographic range expansion of the foraminifera *Amphistegina* in the Mediterranean Sea. Journal of Foraminiferal Research. J Foram Res 42: 235–245.

39) Yokes MB, Meriç E, Avşar N (2007) On the presence of alien foraminifera *Amphistegina* *lobifera* Larsen on the coasts of the Maltese Islands. Aquat Invasions 2: 439–441.

40) Triantaphyllou MV, Koukousioura O, Dimiza MD (2009) The presence of Indo-Pacific symbiont-bearing foraminifer *Amphistegina lobifera* in Greek coastal ecosystems (Aegean Sea, Eastern Mediterranean). Mediterr Mar Sci 10: 73–85.
